# Compulsory Medical Service

**Published:** 1899-01-21

**Authors:** 


### COMPULSORY MEDICAL SERVICE.

There are many medical men who greatly doubt whether the State gives them any just reward for the laborious and expensive course of study which is laid upon them before they are admitted to the Register as qualified practitioners, and when we see the quackery which is rampant on every side, quackery which neither Government nor police make the slightest effort to suppress, notwithstanding that Government maintains (at the expense of the profession) a register of all who, according to law, are duly qualified to practise their profession, we can hardly withold our sympathy from those medical men who consider that they get very little return for all that Government has made them do. What, then, are we to think of the calm impudence of the suggestion made by Mr. Thomas M. Watt, M.R.C.S., that in return for all the manifold advantages (!) conferred by registration every registered practitioner should be compelled by law, as part of the condition of his being registered, to attend any woman in her confinement to whom an "order" has been given by the Guardians. He has a plan for doing away with midwives which is simplicity itself. He would largely extend the Poor Law system of giving orders for midwifery attendance, and he would leave the patients to select their own medical attendant. So far as this goes we do not quarrel with him. But, to make his plan complete, he would make every registered practitioner liable to serve if called upon to attend. What he says is this: "As the medical profession is a monopoly, like the licensed victuallers, created by the State—in the interests indeed of the public, but still, like all monopolies, bringing distinct advantages to those enjoying it—there could, to my thinking, be no just ground of grievance if all members of the profession should be required, as a condition of admission to its benefits and privileges, to engage to honour and act upon these calls of the State, accepting the fees fixed by State regulations, or to pay handsomely to the State year by year for exemption." This is, indeed, a charming prospect, and one cannot but admire the boldness of the man who proposes to levy such a tax upon his compeers. Little as we may be excited by the raids upon the purses of the smaller fry of the profession which are made by medical aid associations and other agencies for undermining their practices, we cannot avoid a shiver of astonishment when we think of Sir John Williams or Dr. Playfair or other mighty practitioners being haled out from their comfortable quarters and made to leave a whole waiting-room-full of patients to attend a 10s. case of pauper midwifery. We have heard a great deal lately about the Irish dispensary doctors, and the "red ticket" which has to take precedence of all other engagements, and thus renders their lives a burden to them; but here is a "red ticket" which is to be laid upon every member of the profession, unless he is ready "to pay handsomely to the State for exemption." We fancy we see the tax-gatherer wandering round Cavendish Square and Harley Street and other select localities collecting his blackmail, and, if the good men are slow in paying up, sending round a few midwifery orders about dinner time to stir them out of their self-content!

